# Incorporating biodiversity responses to land use change scenarios for preventing emerging zoonotic diseases in areas of unknown host-pathogen interactions

**DOI:** 10.3389/fvets.2023.1229676

**Published:** 2023-11-09

**Authors:** Fabio de Oliveira Roque, Beatriz Bellón, Angélica Guerra, Francisco Valente-Neto, Cyntia C. Santos, Isabel Melo, Adriano Nobre Arcos, Alessandra Gutierrez de Oliveira, André Valle Nunes, Clarissa de Araujo Martins, Franco L. Souza, Heitor Herrera, Luiz Eduardo R. Tavares, Mauricio Almeida-Gomes, Olivier Pays, Pierre-Cyril Renaud, Suellem Petilim Gomes Barrios, Lisa Yon, Gemma Bowsher, Richard Sullivan, Matthew Johnson, Carlos E. V. Grelle, Jose Manuel Ochoa-Quintero

**Affiliations:** ^1^Instituto de Biociências, Universidade Federal de Mato Grosso do Sul, Cidade Universitária, Campo Grande, Brazil; ^2^Centre for Tropical Environmental and Sustainability Science (TESS) and College of Science and Engineering, James Cook University, Cairns, QLD, Australia; ^3^BIODIVAG, Univ Angers, Angers, France; ^4^Department of Environmental Science, Rhodes University, Makhanda, South Africa; ^5^Wetlands International Brazil, Campo Grande, Brazil; ^6^Instituto de Investigación de Recursos Biológicos Alexander von Humboldt, Bogotá, Colombia; ^7^Instituto Nacional de Pesquisa do Pantanal, Programa de Capacitação Institucional, Museu Paraense Emílio Goeldi, Cuiabá, Brazil; ^8^Universidade Católica Dom Bosco, Programa de Pós-Graduação em Ciências Ambientais e Sustentabilidade Agropecuária, Campo Grande, Brazil; ^9^REHABS International Research Laboratory, CNRS-Université Lyon 1-Nelson Mandela University, George, South Africa; ^10^School of Veterinary Medicine and Science, University of Nottingham, Nottingham, United Kingdom; ^11^Centre for Conflict and Health, King’s College, London, United Kingdom; ^12^School of Geography, University of Nottingham, Nottingham, United Kingdom; ^13^Department of Ecology, Universidade Federal do Rio de Janeiro (UFRJ), Campo Grande, Brazil

**Keywords:** land-use planning, agriculture, zoonosis, Cerrado, LCLUC, COVID-19 pandemic

## Abstract

The need to reconcile food production, the safeguarding of nature, and the protection of public health is imperative in a world of continuing global change, particularly in the context of risks of emerging zoonotic disease (EZD). In this paper, we explored potential land use strategies to reduce EZD risks using a landscape approach. We focused on strategies for cases where the dynamics of pathogen transmission among species were poorly known and the ideas of “land-use induced spillover” and “landscape immunity” could be used very broadly. We first modeled three different land-use change scenarios in a region of transition between the Cerrado and the Atlantic Forest biodiversity hotspots. The land-use strategies used to build our scenarios reflected different proportions of native vegetation cover, as a proxy of habitat availability. We then evaluated the effects of the proportion of native vegetation cover on the occupancy probability of a group of mammal species and analyzed how the different land-use scenarios might affect the distribution of species in the landscape and thus the risk of EZD. We demonstrate that these approaches can help identify potential future EZD risks, and can thus be used as decision-making tools by stakeholders, with direct implications for improving both environmental and socio-economic outcomes.

## 1. Introduction

Humans have fundamentally transformed ecosystems and shaped the distribution of biodiversity on Earth, mainly through agricultural activities (1), which is expected to continue in the coming years. Food demand is forecasted to increase to meet the needs of the rising human population, estimated to reach 9.73 billion people by 2064 (2). The agricultural intensification and expansion, and the expected continued urbanization and industrialization needed to meet global consumption (3) represent a major threat to biodiversity and conservation in the coming decades. These trends have caused high global concerns in terms of increasing risks of emerging zoonotic disease (EZD) (4). Recent studies have evaluated potential approaches to balance the need for food production and the need for biodiversity conservation and its derived ecosystem services (5, 6). These studies have increased our ability to foresee potential consequences of future land-use trajectories and to develop adapted planning tools, particularly within the framework of the UN Sustainable Development Goals [SDGs; (7)]. Among relevant planning tools, scenario modeling allows to examine how land-use changes may influence biodiversity, food production and provision of ecosystem services under different pathways of future human development and policy choices (8).

With the COVID-19 pandemic, there has been a renewed recognition of the public health risks which arise from direct and indirect interactions between humans and wildlife (4). Indeed, around 70% of Emerging Zoonotic Diseases (EZD), and almost all recent pandemics (e.g., MERS- CoV, Ebola), have been associated with the increased interactions among wildlife, domestic animals, and humans (9), as a result of deforestation, urbanization, and intensification of agricultural systems (3, 10–12). Global changes in the mode and intensity of land-use conversion are expanding the hazardous interactions between people, domestic animals, and the wildlife reservoirs of zoonotic diseases; indeed, wildlife species that harbor higher pathogen loads are more likely to occur in human-managed ecosystems (11). When the rates of habitat conversion are high, the rate of transmission of pathogens between the species inhabiting conserved habitats and those inhabiting converted habitats is expected to be higher (13, 14). Although the mechanisms and landscape contexts that influence the events and rates of transmission of pathogens between wildlife species and humans are not completely understood yet, some operational concepts based on a landscape approach have been recently proposed by Plowright et al. (15), including: 1. the “land-use induced spillover” concept which implies that land-use change can lead to spillover events through a series of complex steps, involving pathogen infection in wildlife and shedding of the pathogen by person-to-person transmission; and 2. the “landscape immunity” concept which implies that some ecological conditions can, when combined, maintain and strengthen the immune function of wild species within a particular ecosystem, while preventing the conditions that lead to high pathogen prevalence and shedding.

In this context, tropical areas are particularly prone to land-use induced spillover, as compared to regions in other latitudes, since they have a larger number and diversity of animal species, as well as a greater number and diversity of their associated pathogens, and they are experiencing rapid land-use changes. This fast land-use conversion is increasing the potential for novel interactions between domestic animals, humans and wildlife, along with their associated pathogens. Indeed, many wild animals cannot survive a drastic reduction in the amount of habitat within a landscape, which could limit access to food and other resources (16, 17), and those that can adapt will increasingly occupy anthropogenic areas, thus increasing the risk of EZD. Evidence also suggests that EZD is related to (i) land-use changes in a given area, (ii) changes in the interaction networks of different species driven by native vegetation loss, (iii) local hunting practices, (iv) changes in behavior of pathogen vectors, and (v) the sharing of newly altered landscapes by people, domestic and wild animals, and insect vectors of disease (15, 18–20). Therefore, it is vital to understand how different land-use scenarios might affect species distributions. This can then aid in the identification of potential future EZD risks, which can be used to inform landscape management policies (16, 21).

Based on these outlined assumptions and concepts, we explored potential land-use scenarios to reduce EZD risks using a landscape modeling approach. It is important to highlight that our study was designed to only address strategies for cases where the dynamics of pathogen transmission among species are poorly known and the concepts of land-use induced spillover (LUIS) and landscape immunity (LI) can be used very broadly. First, we projected changes in the native vegetation cover, as a proxy for habitat, under three potential scenarios: (a) business as usual (BAU), (b) avoidance of LUIS, and (c) LI. We then evaluated the effects of the proportion of native vegetation cover on the occupancy probability of mammalian species (including groups that are likely to harbor human-shared pathogens, such as Artiodactyla and Rodentia), and we analyzed how the different land-use scenarios might affect the distribution of species in the landscape, and thus the risk of EZD. We developed our analysis on a region at the interface between the Brazilian Cerrado and the Atlantic Forest, areas recognized as biodiversity hotspots facing strong agribusiness expansion and infrastructural development (22). The rapid land-use changes in this region can lead to local extinctions, increased human-wildlife conflict, and result in loss of ecological services. Therefore, our study area can serve as a good model to understand how land-use changes could be related to the emergence of EZD and how landscape modeling frameworks such as the one presented can aid in identifying strategies that reconcile the potentially conflicting objectives of supporting both agricultural production and biodiversity conservation in tropical regions.

## 2. Materials and methods

### 2.1. Study area

**Figure 1:**
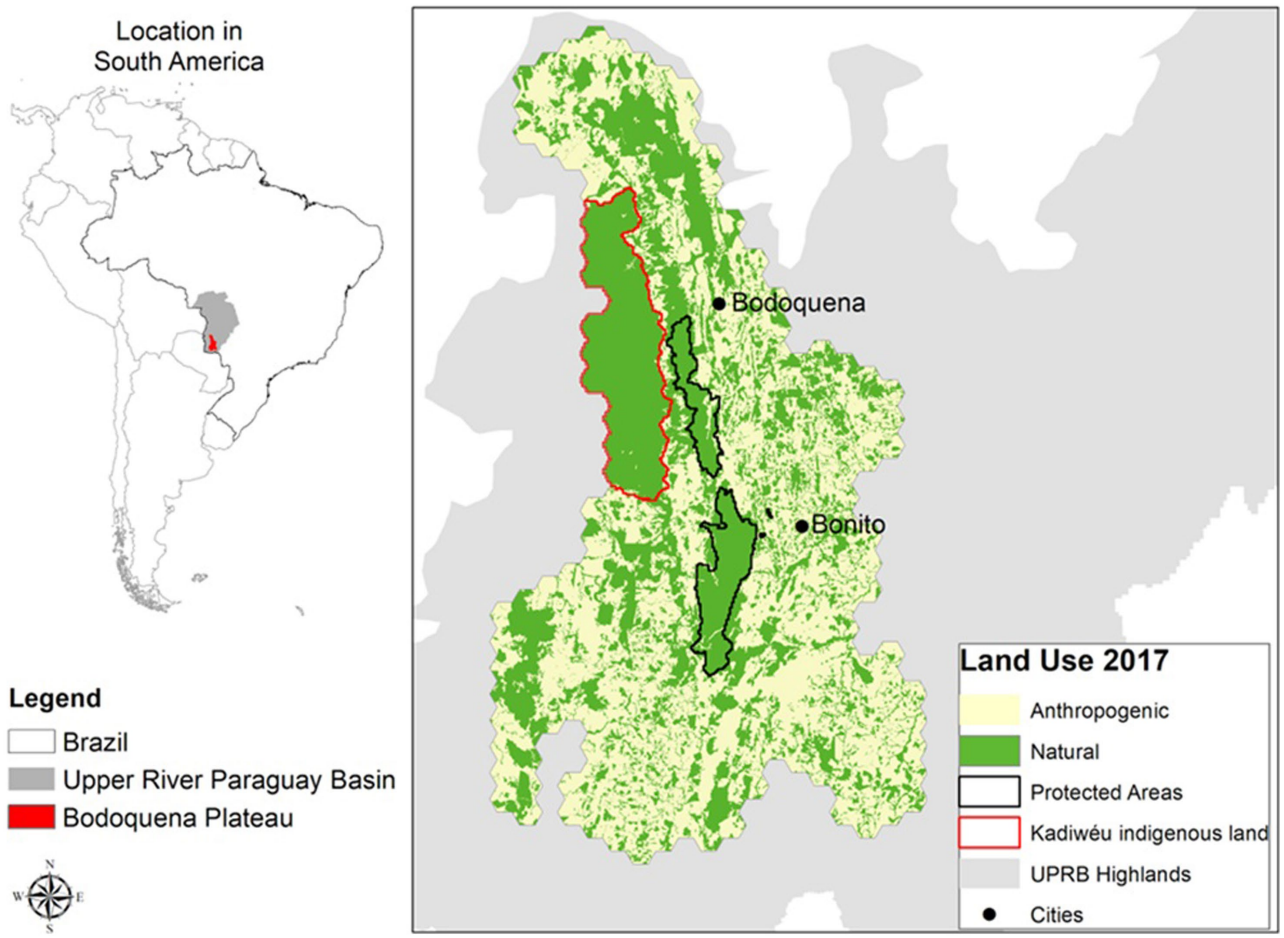
The study region, covering 18.000 km^2^ in the centre of South America, included the municipalities of Jardim, Bela Vista, Caracol, Corumbá, Miranda, Porto Murtinho, Bodoquena, and Bonito in Mato Grosso do Sul state, Brazil. The study area was divided into 1 km^2^ hexagons on which different modelling scenarios of land use change were applied (introduced in section 2.2). The base land use data was recovered from SOS Pantanal et al. (23) and reclassified to two general classes: Native vegetation, and Anthropogenic land use.

Our study focused on the Bodoquena Plateau and its surrounding areas, over an extension of 18,000 km^2^ (Figure 1). This region is part of the Upper Paraguay River Plateau, where more than 60% of its native vegetation has been converted into agricultural lands over the last 50 years (24). Two of the world’s most threatened biodiversity hotspots, the Atlantic Forest and the Cerrado, converge in this area. In addition, this region holds one of the most extensive karstic systems in Brazil and is one of the most important eco-tourism attractions worldwide, with crystal clear waters, caves, dolinas, and underground rivers. The Bodoquena National Park is a c.700 km^2^ central region within the Bodoquena Plateau which is surrounded by human-modified landscapes (see Figure 1), mostly by pastures for cattle ranching, but also by soybean and maize plantations. Part of the study region is also occupied by the indigenous Kadiwéu territory, which, in total, comprises 1,519 km^2^.

### 2.2. Species occupancy responses to the proportion of native vegetation across the landscape

In order to understand how current and future potential land-use changes, across three scenarios, could affect the occupancy of the mammal species in our study area, we estimated the species occupancy responses to the proportion of native vegetation across the landscape.

The occupancy response of 29 medium to large-sized mammal species to a gradient of native vegetation cover in the Bodoquena Plateau was estimated using the hierarchical multi-species occupancy model based on Bayesian Markov Chain Monte Carlo-based inference (25) presented by Bellón et al. (26). Mammal species were selected because they are associated with ecological functions and services which are crucial for many other species and they are one of the most sensitive groups to land-use changes, especially in the tropics (27). The 29 species selected for our study (Table 1) were all the mammal species detected during a camera trap survey carried out during 2017, in which 189 camera traps were deployed across the study area [further details on the sampling strategy may be found in (26)]. Bellón et al. (26) found these species responded differently to vegetation gradients in the region and some of the species, such as those belonging to the Artiodactyla, Rodentia and Primates groups which are likely to harbor human-shared pathogens (9).

**Table 1:** List of mammal species.

Order	Family	Species	English common names	Zoonotic host status	Trend
Artiodactyla	Cervidae	*Mazama americana*	South American Red Brocket	Non-host	++
Artiodactyla	Cervidae	*Mazama gouazoubira*	South American Brow Brocket	Host	+
Artiodactyla	Tayassuidae	*Pecari tajacu*	Collared Peccary	Host	++
Artiodactyla	Tayassuidae	*Tayassu pecari*	White-lipped Peccary	Non-host	++
Carnivora	Canidae	*Cerdocyon thous*	Crab-eating Fox	Non-host	- -
Carnivora	Canidae	*Chrysocyon brachyurus*	Maned Wolf	Non-host	0
Carnivora	Felidae	*Herpailurus yagouaroundi*	Jaguarundi	Host	0
Carnivora	Felidae	*Leopardus pardalis*	Ocelot	Non-host	++
Carnivora	Felidae	*Panthera onca*	Jaguar	Host	0
Carnivora	Felidae	*Puma concolor*	Puma, Cougar	Host	-
Carnivora	Mustelidae	*Eira barbara*	Tayra	Host	++
Carnivora	Procyonidae	*Nasua nasua*	South American Coati	Host	+
Carnivora	Procyonidae	*Procyon cancrivorus*	Crab-eating Raccoon	Non-host	0
Cingulata	Chlamyphoridae	*Cabassous unicinctus*	Southern Naked-Tailed Armadillo	Non-host	0
Cingulata	Chlamyphoridae	*Euphractus sexcinctus*	Six-banded Armadillo	Non-host	-
Cingulata	Dasypodidae	*Dasypus novemcinctus*	Nine-banded Armadillo	Host	- -
Didelphimorphia	Didelphidae	*Didelphis albiventris*	White-eared Opossum	Host	0
Didelphimorphia	Didelphidae	*Gracilinanus agilis*	Agile Gracile Opossum	Non-host	0
Lagomorpha	Leporidae	*Sylvilagus brasiliensis*	Tapeti	Non-host	++
Perissodactyla	Tapiridae	*Tapirus terrestris*	South American Tapir	Non-host	+
Pilosa	Myrmecophagidae	*Myrmecophaga tridactyla*	Giant anteater	Host	- -
Pilosa	Myrmecophagidae	*Tamandua tetradactyla*	Southern Tamandua	Host	+
Primates	Cebidae	*Sapajus cay*	Hooded Capuchin	Non-host	0
Rodentia	Caviidae	*Hydrochoerus hydrochaeris*	Capybara	Host	0
Rodentia	Cricetidae	*Hylaeamys megacephalus*	Azara’s Broad-headed Rice Rat	Host	0
Rodentia	Cricetidae	*Rhipidomys macrurus*	Long-tailed Climbing Mouse	Non-host	0
Rodentia	Cuniculidae	*Cuniculus paca*	Agouti, Spotted Paca	Host	+
Rodentia	Dasyproctidae	*Dasyprocta azarae*	Azara’s Agouti	Non-host	++
Rodentia	Echimyidae	*Thrichomys pachyurus*	Paraguayan Punaré	Non-host	0

Scientific names were recovered from Abreu et al. (28). English common names were recovered from IUCN (29). Each species was assigned to one of the two categories of zoonotic host status: either “Non-host” if they harbour no known zoonotic viruses or “Host” if they are known to harbour one or two zoonotic species. The information used to classify the species according to their number of known zoonotic viruses hosted was obtained from the supplementary materials of the review of Johnson et al. (14). The column “Trend” is related to changes in occupancy across the deforestation gradient: “++” = high increase in occupancy probability with an increase in % native vegetation, “+” = increase in occupancy probability, “--” = high decrease in occupancy, “-” = decrease in occupancy, “0” = no response.

In order to estimate the occupancy response of mammal species to the percentage of native vegetation in the study area, we used the mammal occurrence dataset and an occupancy covariate representing the percentage of native vegetation within a 1km^2^ hexagonal grid across the study area derived from a land use/cover map from 2017 (Figure 1). Following Bellón et al.’s (26) modeling framework we used the R package NIMBLE (Numerical Inference for statistical Models for Bayesian and Likelihood Estimation) (30, 31) and formulated our model to account for imperfect detection by modeling both the species occupancy probability and the detection probability (32). Details on the methods used to parameterize the models and evaluation of model convergence may be found in (26).

### 2.3. Scenarios

We used the same land use/cover map (Figure 1) used in the species occupancy model presented in the previous section (section 2.2) as the base layer from which we projected three native vegetation change scenarios: the “business-as-usual” (BAU) scenario, the “avoidance of land-use induced spillover” (ALUIS) scenario, and the “landscape immunity” (LI) scenario.

In the “BAU” scenario, we projected native vegetation loss by extrapolating the trend of recent years (2008–2016) (23) and assumed the full implementation of the Brazilian environmental legislation (Native Vegetation Protection Law - NVPL, known as the Forest Code). The Brazilian Forest Code (FC) aims to limit natural vegetation conversion and protects valuable ecosystems in private properties. Revised in 2012 by the law #12615/2012 (33), the FC requires landowners within the administrative boundaries of the Cerrado biome to maintain natural vegetation in so-called “legal reserves” on at least 20% of their property, and also delimits areas of permanent protection for specific ecosystems, in particular around watercourses and in areas of steep slopes. The percentage of native vegetation was modeled within the 1 km^2^ hexagons (represented in Figure 1), which we chose as a proxy of average rural property size.

**Figure 2:**
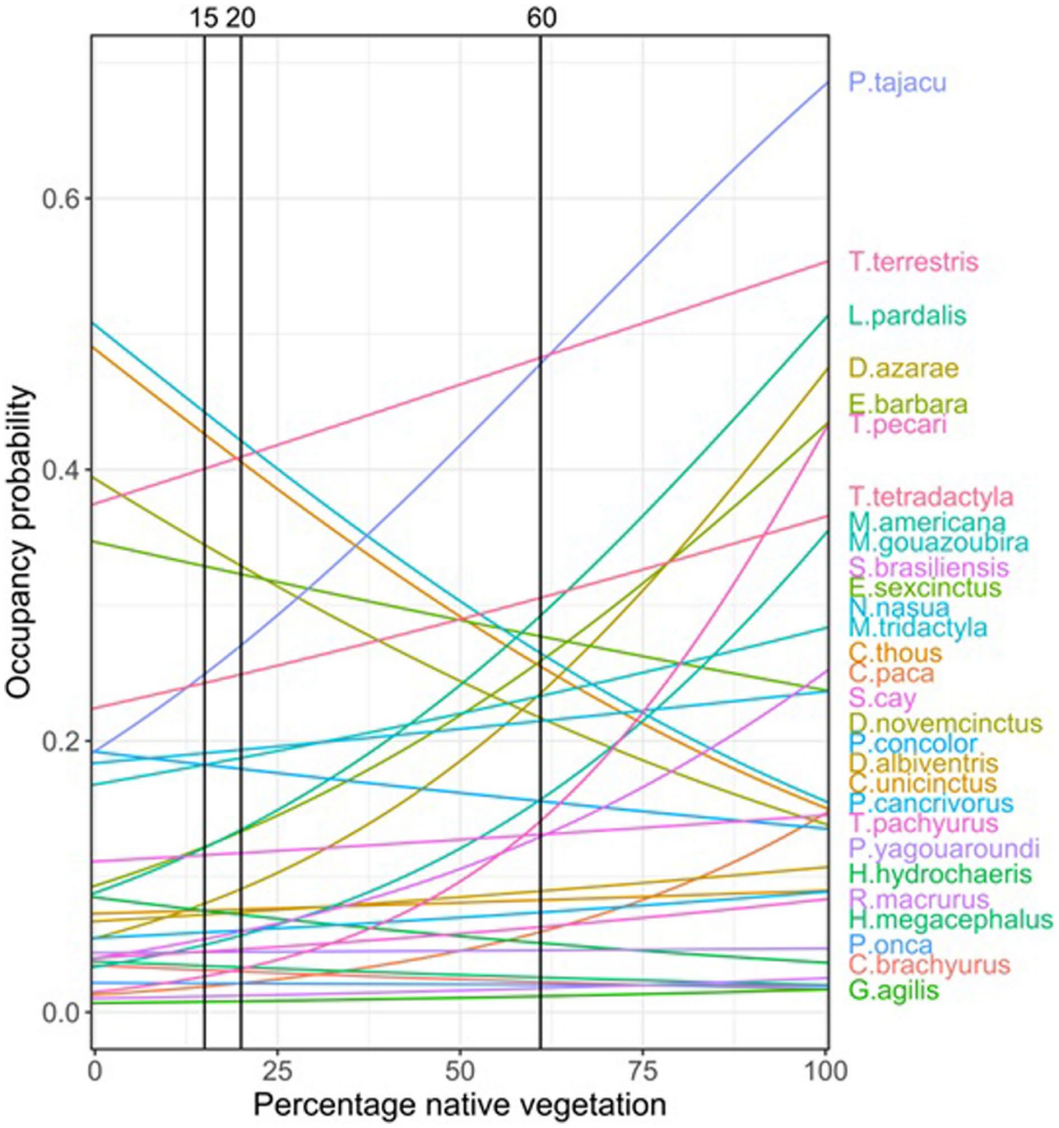
Current and projected land use for 2050 according to “business-s-sual” (BAU) and “avoidance of land use induced spillover” (ALUIS) scenarios.

The ALUIS scenario assumes that spillover events occur when a reservoir host species (e.g., mammalian species) comes into contact with a novel host species (e.g., humans). The pathogen may be transmitted from the reservoir population to a novel host species and may then be transmitted within the novel host population. In the ALUIS scenario, we considered the same land-use trends assumed in the BAU scenario, with the addition of the creation of 5 km^2^ buffer zones around protected areas, indigenous lands, and remnants of native vegetation of more than 5,000 ha, as well as within 150 m from the riverbank (as established by State Law #1871/1998). This choice was made considering that protected areas are a cornerstone of biodiversity conservation (34), and buffer zones can help prevent contact between animals and humans in the surrounding agricultural lands. Additionally, most mammalian species in the region respond to changes in occupancy related to vegetation cover in a smaller landscape extent (35). Within these buffer zones, we kept the minimum proportion of native vegetation that the FC requires to be maintained in private rural properties in so-called “legal reserves” (i.e., 20% in the Cerrado biome). It is important to note that 20% of legal requirements is close to the 15–20% thresholds we set for the less sensitive species in the gradient of native vegetation cover (Figure 2). As such, in our scenario, the buffer zones act as an ecological barrier for most species between protected areas and agricultural/rural lands.

The LI scenario is based on evidence that the dilution effects are associated with high levels of biodiversity (15, 36). We considered the same trends assumed in the BAU scenario, except that the landscapes maintain a percentage of native vegetation above the threshold for most species (60% as a proxy of the whole community; Figure 2).

All scenarios were conservative and pragmatic in terms of environmental change assumptions. We assumed that: (i) the current environmental laws will be implemented, (ii) no new protected areas will be created, and (iii) the rate of land conversion will not be higher than that experienced in the last 15 years.

### 2.4. Scenario building

In order to understand how current and future land-use changes may relate to changes in the selected species occupancy, we created future land use maps, and included the habitat threshold values (i.e., 15 and 60%) into scenario building (Figure 2). These two values mark the points where the occupancy probability, which is mostly influenced by native vegetation, showed changes for most species. We produced maps for two different years: 2017 (the year of our mammal occurrence dataset) and 2050 (the target date for the Post-2020 Global Biodiversity Framework, https://www.cbd.int/gbo).

For 2017, we used the native vegetation layer (Figure 1) to interpolate the occupancy thresholds over the study area at a 1 km spatial resolution. For 2050, we used a dynamic and spatially-explicit model, developed by Rosa et al. (8), that estimates the magnitude and location of future native vegetation loss using a set of drivers of vegetation loss derived from peer-reviewed literature. The model uses Monte Carlo Markov Chains (MCMC) to return for each parameter a posterior probability distribution, from which we can extract the posterior mean and a range of credibility, given the structure of the model and the data used for calibration. We used four 2-year intervals to perform the analyses (2008–2010, 2010–2012, 2012–2014, and 2014–2016). For each time step, binary maps of change were produced (1: native vegetation, 0: anthropogenic), which were then integrated based on the model’s 100 iterations (sampling of later distributions) to determine the overall probability of change. These steps were repeated for each of the four time periods, as the model will project future conversion based on observed rates of change, and these had different rates of change. Once all models were calibrated, the best (with the combination of variables that produced the highest probability of testing) was used to project the future probability of native vegetation loss by 2050. The cumulative probability of conversion in 2050 was determined for each model individually (models 2008–2010, 2010–2012, 2012–2014, and 2014–2016), as well as based on a set of all model outputs (i.e., integrating all projection models made for that year). By spatializing the probability of loss of vegetation for the UPRB in each scenario, we obtained estimates of probability of vegetation loss by 2050 at a 1 km resolution across the study area, which allowed us to identify the areas with the highest probability of vegetation cover loss. This modelling framework is fully described by Guerra et al. (37), where it was tested in the Upper Paraguay River Basin, a region that partially spatially overlaps our study area.

### 2.5. Coupling occupancy thresholds and land use scenarios

For the ALUIS scenario, the threshold for the most sensitive species (set at 60%) was used for the 1 km^2^ cells within protected areas, indigenous lands, remnants of native vegetation with more than 5,000 ha, and Permanent Protection Areas (PPA), i.e., a strip of native vegetation around rivers which is protected by the Forest Code. For the 1 km^2^ cells within the buffer around these protected areas we used the native vegetation threshold value of the least sensitive species (15%), and for the cells in other areas, we used the 20% native vegetation value, according to the requirements of “legal reserves” established in the Forest Code. In this scenario, the area in need of restoration was calculated within protected areas, indigenous lands, and PPA, as well as in their buffer areas, by assessing locations with native vegetation coverage between the two selected thresholds (60 and 15%).

For the LI scenario, the threshold value of the most sensitive species (60%) was used for protected areas, indigenous lands, remnants of native vegetation with more than 5,000 ha, and the PPA, and 20% was used for all other areas.

Finally, in order to evaluate the amount of restoration of native vegetation required under each scenario to achieve the aim of reducing EZD risk, the occupancy threshold results in 2050 were projected to predict the vegetation cover above and below occupancy thresholds.

## 3. Results

### 3.1. Species responses to land-use changes

Most of the 29 species analyzed responded positively to the increase of vegetation cover percentage, especially those that are highly dependent of resources from native vegetation (such as fruits, and shelters). Among those species it is relevant to mention that *P. tajacu*, *T. terrestris*, *L. pardalis*, *T. pecari*, and *M. americana* showed a clear positive response on their occupancy probability across the gradient of native vegetation cover. For some species, although vegetation cover is a relevant variable, the response on the occupancy models was not as important. However, for a few species the increase in vegetation cover was related to a decrease on the probability of occupancy such as *P. cancrivorous* or *E. sexcinctus*; those species seemed to find a benefit from native vegetation loss across the gradient. It is important to highlight that among the most sensitive species to vegetation cover changes, there are some species of rodents (e.g., *H. hydrochaeris*), and Artiodactyla (e.g., *P. tajacu, T. pecari*) that showed important changes in their occupancy as a response to habitat loss in the landscape (Table 1). Some of the species of these groups, such as Rodentia and Artiodactyla, are known to carry high numbers of zoonotic virus (14, 38). Those species increasingly occupying deforested areas are also shorter-lived species for which high infectiousness and consequently probability of transmission is most likely to take place (38).

### 3.2. Land-use scenario modeling

**Figure 3:**
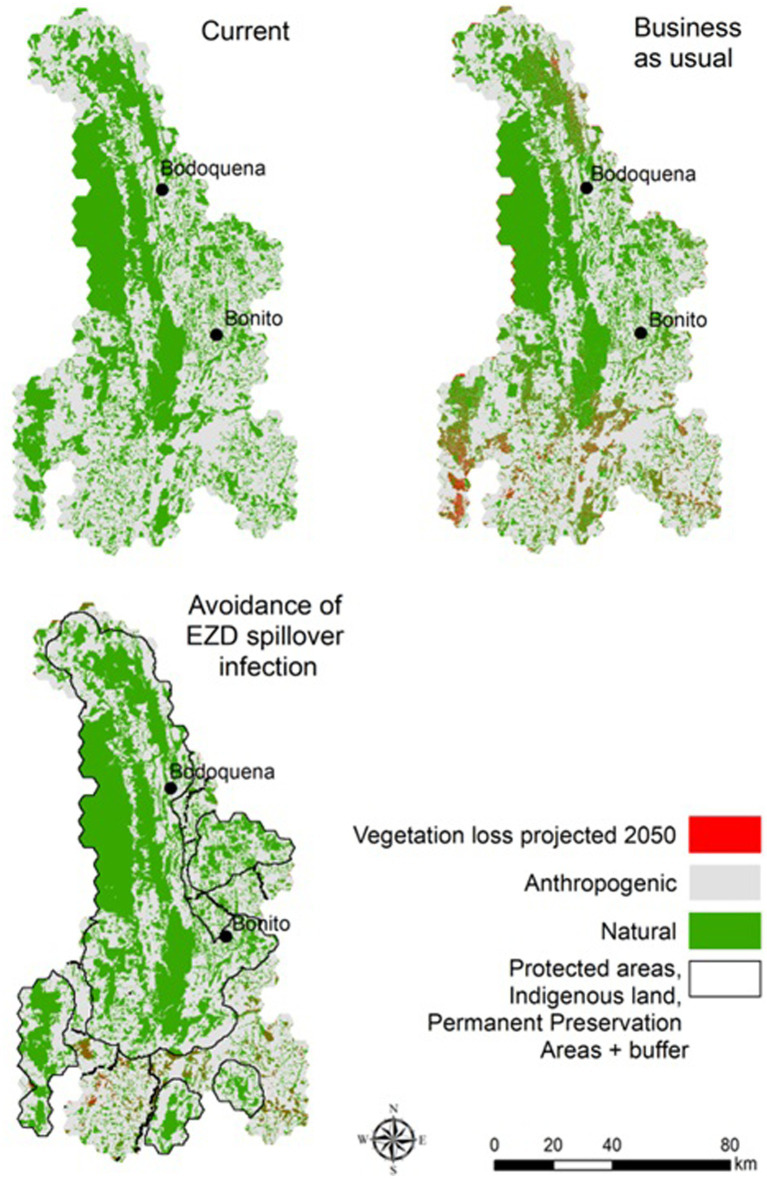
Native vegetation below and above legal values (Forest Code – FC) and thresholds according to each scenario.

The results indicate that implementing the ALUIS scenario would reduce native vegetation loss across the region during the coming years. According to our land-use models, the BAU scenario predicts a loss of 597 km^2^ (±18.9, 95% CI) of native vegetation by 2050. The ALUIS scenario, by contrast, predicts a loss of 275 km^2^ (±8.0, 95% CI) of native vegetation by 2050 (54% less than BAU; Figure 3).

**Figure 4:**
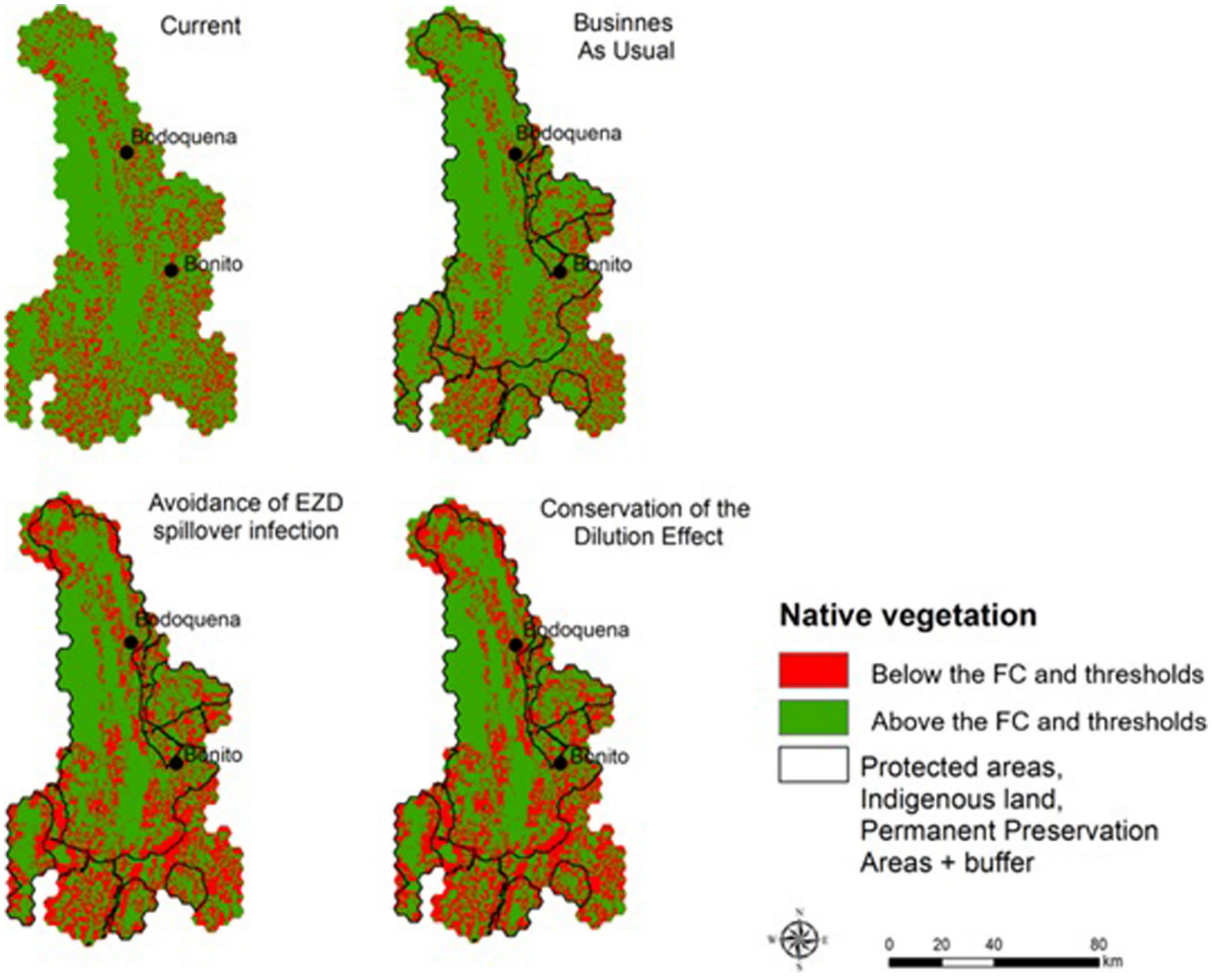
Areas that need to be restored in order to maintain native vegetation within the proposed native vegetation limits. The values represent the % of native vegetation that needs to be restored in each 1 km^2^ hexagon.

**Figure 5:**
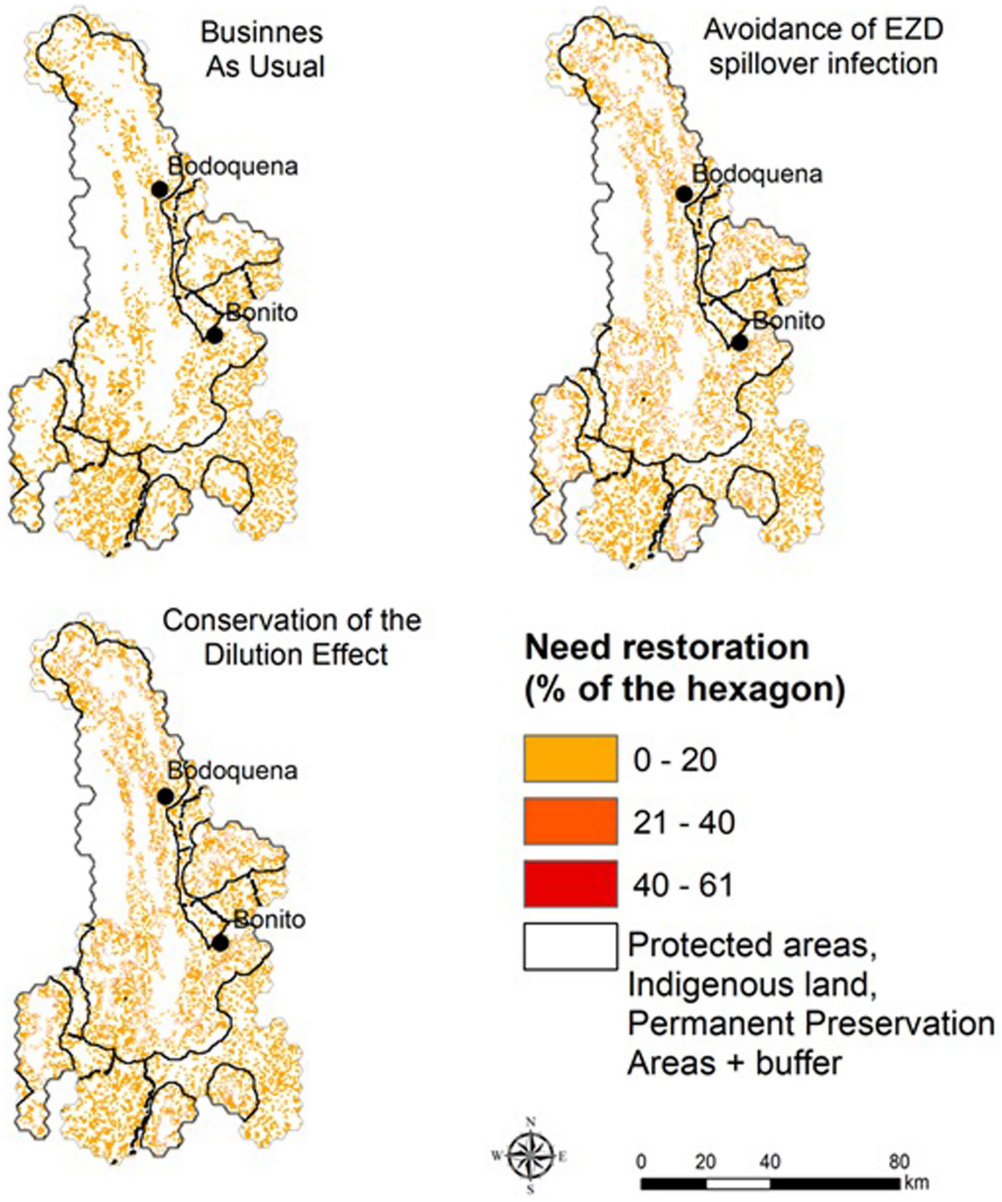
Categories of native vegetation cover and probability of occupation of large birds and mammals on the study area. The column with of each species is the average probability of persistence of each species in each category of native vegetation amount and height of each column is the sum of the probability of each of the species according to the modeling approach used in the paper.

In the scenarios projected for 2050, we calculated the area that would need to be restored to reach the minimum targets for native vegetation based on the proposed thresholds for each scenario (Figure 4). In the BAU scenario, 846 km^2^ must be restored, in the ALUIS scenario a minimum of 965.2 km^2^ is needed, and in the LI scenario, restoration of 1,007.6 km^2^ is required (Figure 5).

## 4. Discussion

An unprecedented number of studies have been undertaken to reconcile efforts in food production, safeguarding of nature, and protection of public health [e.g., (4, 39–41)]. However, to date, there has been little to identify an approach to harmonize these different, and sometimes conflicting targets using empirical data. Our results showed that combining information on current and modeled land-use changes may be a strategy to reduce EZDs outbreaks based on appropriate biodiversity management. Proposed future scenarios based on empirical information on land-use change allowed us to explore and assess the implications of managing EZDs, considering both assumptions from the “land-use induced spillover” and the “landscape immunity” frameworks introduced by Plowright et al. (15), with implications for biodiversity protection, agriculture, and restoration in the region, particularly for regions under limited known pathogen dynamics landscape, such as the Bodoquena Plateau in Brazil.

Our results showed how changes in the amount of vegetation cover might influence the occupancy probability of different species of mammals across deforestation gradients. For most of these particular species their category of zoonotic disease status is unknown, nonetheless, and according to several authors (see (38) for a review), aspects related to their order might inform on their potential status such as rodents known to be the group with the larger proportion of zoonotic diseases per species with at least one potential zoonotic virus. This information, together with the assumption proposed by Faust et al. (13), in which an intermediate proportion of habitat converted may have an impact on the risk of transmission on the whole community, allows us to draw some suggestions about the relevance of using land-use change scenarios as a strategy to reduce the potential of zoonotic disease outbreaks.

The implementation of any EZD strategy proposed here would reduce native vegetation lost by 2050. Under the BAU scenario, there would be an increase in the proportion of the landscape with native vegetation cover under the occupancy threshold values, implying a restoration effort of 846 km^2^. Nonetheless, our results have clear practical implications of the implementation of the ALUIS scenario and the LI scenario. For example, our study highlights the need for an increase in restoration targets under the ALUIS and LI scenarios that are similar to the restoration efforts needed in the BAU scenario, but slightly higher (965.2 and 1,007.6 km^2^ respectively).

Planning and managing landscapes to manage the populations of pathogen hosts and vectors, as we proposed here, can be considered an area-based management strategy for preventing future zoonotic epidemics (39). The ALUIS scenario assumes that pathogen transmission between species inhabiting conserved and converted habitat is highest when rates of habitat conversion are intermediate (13–15). In our case, in the intermediate category of native vegetation coverage (i.e., between 60 and 15%) most mammalian species show a variation on the probability of occupancy across the gradient of remaining vegetation cover. It implies that around this level of native vegetation coverage, we can see the most interactions (co-occurrence) between forest-dependent (losers) and habitat generalist (winners) species (Figure 5). Although controversial, a potential consequence of this pattern is that more species with fast-paced life-history strategies will replace those with slower-paced life histories as host communities become fragmented or disturbed, and biodiversity loss is fast or abrupt. This could result in an increase in host exposure to parasites by shifting host behavior (36). However, an ongoing question is how to best define this intermediate category. In the case of managing the dilution effect, a key strategy might be to conserve the whole community of hosts and vectors in a landscape. Here a key question, with a long history in conservation ecology is: how much native vegetation should be conserved to maintain the whole community and ecological processes in a given area? Both the ALUIS and the LI scenarios require information about the response of hosts, vectors, and associated pathogens to land-use changes in a region which are not available for most tropical regions, including the region we studied. To overcome this challenge, we expanded the idea of using an ecological threshold as a limit of land-use changes (21, 42). In this context, we propose the inclusion of levels of host thresholds to land-use changes as proxies to define ALUIS and LI scenarios, in other words “safe operating landscapes for EZD avoidance” (43).

Thus, for defining “safe operating landscapes for EZD avoidance” in any region, development at the landscape scale should involve a process of co-design of plans with all the relevant stakeholders, and with specific objectives and thresholds designated (44). As such, the scenarios proposed in our study should not be seen as the most plausible scenarios, but instead encompass a number of potential projected futures (45) that should be collectively discussed, considering levels of risks, and cost-benefits for people, animals and ecosystems, in the coming years. With this in mind, the costs of the interventions and strategies based on different “thresholds of the hosts” could be more rationally assessed. This assessment must include not only the direct costs of an intervention (e.g., restoration), but also changes in future income (opportunity costs) for different stakeholder groups, as well as analysis of cost-benefits for monitoring and prevention actions (e.g., avoiding deforestation) (46). Each scenario implies different strategies, including governance, trade-offs between agricultural lands and protected areas, different cost-benefits for relevant stakeholders, and uncertainties. For example, in our study case, the BAU scenario will clearly result in larger areas for food production in the region, however, it would also result in a larger decrease in the surface of native vegetation, resulting in levels below the thresholds allowing the occurrence of selected indicator species (e.g., more than 846 km^2^ will be below the threshold for the most sensitive species such as *P. tajacu*). This will decrease the possibility of a dilution effect and increase chances of spillover. On the other hand, if decision-makers choose to go towards the ALUIS scenario, the restoration required to reach the proposed native vegetation thresholds would be around 965 km^2^ which clearly will imply economic costs, while in the LI scenario, the value will be 4.3% more. Thus, the benefits and costs to different stakeholder groups (farmers, politicians, tourists, the environment) in the future are highly variable, depending on the desired outcomes, and thus the desired scenario. However, decisions about which scenario to select as a target should be determined with inputs from a range of actors in the region.

Area-based management as a single strategy is not enough to build “safe operating landscapes for EZD avoidance” (15, 39). It is critical to consider the linkages that connect food production, conservation, and pathogens, including new relationships between wild and domestic animals, hunting activities, agricultural practices, trade, transport, markets, and food consumption patterns. In fact, we should also consider the socioeconomic and environmental interactions over distances (telecoupling process) as relevant aspect on the implementation of safe operating spaces. Petrovan et al. (39) proposed more than 150 recommendations for preventing future zoonotic epidemics. Many of them directed to farmers, the tourism sector, and governments. For instance, hunting is a context-dependent variable which is not currently evaluated in Bodoquena Plateau, but given its clear importance for potentially driving future outcomes in terms of EZD, it should be considered in “safe operating landscapes for EZD avoidance” plans for our study area. Other strategies that should be contemplated include: (i) developing certification for registered farms that demonstrate enforcement of guidelines on safe production standards; (ii) introducing legislation to reduce and control the spread of animal agriculture and overlap/proximity to tropical forest/undisturbed ecosystems; (iii) conduct risk assessment at the wildlife–livestock–human interfaces to inform the type of emergency response or longer-term planning for prevention and control of zoonotic pathogens. However, it is important to highlight that the implementation of any strategy to reduce the risk of zoonotic epidemics should be decided collectively and discussed by the different sectors, such as cattle ranchers, and should be more closely studied, monitored and reported. For example, environmental police and state government programs could adopt a preventive approach, informing the public about the health of wildlife species killed through illegal hunting or found dead on the roadside. Moreover, monitoring the movement and behavior of animals can bring information on the frequency of invasion of cultivated lands and, consequently, the likelihood of increased contact with humans (13). For example, in the Bodoquena Plateau, peccaries are the most frequent wildlife species seen in crop fields around protected areas and are generally responsible for some crop damage. Understanding interspecies contact is key for studies of landscape spillover, and these will be reflected in seasonal and interannual epidemics (47). Therefore, determining the frequency of human or livestock interactions with different wildlife taxa, and the health status of these species, could be seen as a key strategy to guide policy interventions to minimize public health risks and safeguard animal health in the future. The SISS-Geo (sissgeo.lncc.br), a free platform, available on smartphones and on the web, for monitoring the health of wild animals in natural, rural and urban environments, is a promising system in the direction to improve EZD monitoring in Brazil. To integrate and operationalize these ideas, we also strongly support the suggestion by Patz et al. (12) about the importance of creating “Centers of excellence in ecology and health research and training,” based on a network of international and regional universities and/or research institutes, with close links to the surrounding communities. More broadly, we agree with many studies which suggest the implementation of the “One Health” approach to integrate all health centers and health professionals [e.g., (48, 49)] in a collective effort. For the region of the Bodoquena Plateau, we also suggest these initiatives should explicitly include agriculture as a critical issue, and thus suggest the implementation of: “Centers of excellence in biodiversity and ecological services, food production, and health research and training.”

In terms of modeling and datasets, we made a number of assumptions that should be taken into account in future studies. First, some responses of zoonotic disease to land-use changes could be idiosyncratic (50), and not all species present clear thresholds respond non-linearly to land-use change. In these cases, the scenarios and management strategies that should be considered, the model built for each species separately, as well as the limits for acceptable risk, should be decided based on information and perception of risks from relevant stakeholders and societal contexts. Second, in terms of biodiversity responses to landscape dynamics, our model only considers the thresholds for native vegetation cover. The functional role of the landscape matrix, potential corridors, and the habitat configuration in shaping biodiversity, remains largely unknown in the region. Agricultural practices are another aspect to be considered because they dramatically modify the landscape and have an effect on species’ occupancy (26). Third, our scenario approach does not evaluate global demands for food, changes in the demand for certain types of food, such as plant-based products, which can drive important changes in the agricultural systems. This evaluation is significant for future studies because international food demands can play a critical role in the dynamic of land-use changes in regions, which can be particularly affected by commodity production, such as soy and beef (51). Fourth, our modelling process does not explicitly consider human social interactions, adaptation, and changes in people’s behavior and movements across the landscapes, which are key for the future of this region (52), which in 2022 received more than 200 thousand tourists.

### 4.1. Concluding remark

Our proposal is not a panacea to designing future landscapes, but it should be seen as a first step to incorporate ecological and distributional information of the hosts as proxies to build safe operating landscapes or keep landscape immunity in areas where pathogens and their dynamics are poorly known. However, at least, in regions where the ecology of the diseases is not well understood, focusing on managing and conserving natural habitat should be seen as a precautionary and reasonable first step to reduce general risk of transmission of multiple pathogens, even if this strategy is not the single most efficient control method for individual diseases (53). In this context, our spatially- explicit framework is useful to visualize general land-use tradeoffs and synergies, but much work remains to translate them into a regional planning tool. In this way, we suggest that our approach should be part of the analytical toolbox for designing new landscapes in tropical regions because it helps to for see the challenges in a spatially-explicit and transparent way. It is particularly useful in the first steps of building participatory-decision processes in regions where information on biodiversity and EZD responses to land use are poorly known.

## Data availability statement

The original contributions presented in the study are included in the article/supplementary files. Further inquiries can be directed to the corresponding author.

## Author contributions

FOR and JMO-Q conceived and formulated the idea. BB, FVN, FOR and AG developed the mathematical models. CCS, IM, ANA, AGO, AVN, SPGB and JMO-Q data collection. FVN, BB, AG and JMO-Q analyzed the data. All authors contributed to the article and approved the submitted version.
